# Responses of Persian walnut on foliar applications of different biostimulants

**DOI:** 10.3389/fpls.2023.1263396

**Published:** 2023-10-17

**Authors:** Gergely Simon, Géza Bujdosó, Miljan Cvetkovic, Ozan Tevfik Alp, Laurine Kithi, Richárd Oláh, Gitta Ficzek, György Végvári

**Affiliations:** ^1^ Department of Fruit Growing, Institute of Horticultural Sciences, Hungarian University of Agriculture and Life Sciences, Budapest, Hungary; ^2^ Research Center of Fruit Growing, Institute of Horticultural Sciences, Hungarian University of Agriculture and Life Sciences, Budapest, Hungary; ^3^ Faculty of Agriculture, University of Banja Luka, Banja Luka, Bosnia and Herzegovina; ^4^ Aro-Peritum Ltd., Diósviszló, Hungary; ^5^ Faculty of Natural Sciences, Institute of Viticulture and Oenology, Eszterházy Károly Catholic University, Eger, Hungary

**Keywords:** HPLC, inner content value, kernel, phenolic compounds, nut characteristics

## Abstract

Biostimulants have different effects on plants. The aim of this paper is to determine responses of the ‘Alsószentiváni 117’ walnut cultivar on foliar applications of different biostimulants (Wuxal Ascofol, Kondisol, Alga K Plus). The nut traits (nut length, nut diameter, nut weight, kernel weight) and some phenolic compounds of the kernel were measured and detected. In 2020, during warmer early spring weather conditions under pistillate flowering receptivity, chlorogenic acid and quercetin content of kernels treated with Kondisol were higher than in control. All biostimulants influenced positive effects on catechin and rutin content, as well as treatments made with Wuxal Ascofol and Kondisol increased the juglon content of the kernel. In 2021, when the spring weather was typical for that period, only the Kondisol treatments had increasing effects on the catechin and chlorogenic acid content, than the control. The rutin and quercetin concentrations reached the highest value in this trial by Alga K Plus applications. The juglon content decreased in this year compared to the control. The pirocathecin, cinnamic acid, and gallic acid (except Wuxal Ascofol treatment in 2021) content decreased in all treatments in both observed years. Responses of woody fruit species on biostimulants applications depend on the weather conditions. Biostimulants had positive effects on the nut size characteristics in both observed years.

## 1 Introduction

There is a keen interest in nut tree crops worldwide, because their production shows a massive increasing trend in many countries. During the last two decades the global Persian or English dried walnut (*Juglans regia* L.) production increased by 20% annually. Currently, the harvested quantity of dried walnuts with shells is 3.5 million tons globally (FAO, 2023). In Hungary, the ecological demands of walnut are similar to wine grape (*Vitis vinifera* L.) (Nyitrainé Sárdy et al., 2022) and apricot (*Prunus armeniaca* L.) (Mendelné Pászti et al., 2023). The growing area doubled to 6,000 ha during the past three decades, and the production reached the 6,000 tons dried shelled nuts annually (FAO, 2023).

The cultivars with large, at least 32 mm in diameter nut size are the most valuable product on the market (Akca, Y et al., 2016; Vahdati et al., 2019; Iordănescu et al., 2021; Özcan et al., 2022; Sokolova, 2022; Sutyemez et al., 2022; Paunović and Rade, 2023). There are some possibilities for growers to increase the nut size and to enrich the phenolic compounds of kernels. Among the phenolics, ellagic acid, ferulic acid, gallic acid, catechin, vanillic acid, caffeic acid, sinapic acid, salicylic acid, rutin, and epicatechin were abundant in the kernel and seed coat (Colaric et al., 2005; Bujdosó et al., 2014; Gharibzahedi et al., 2014; Kónya et al., 2015; Trandafir et al., 2016/a; Rahmani et al., 2018; Kafkas et al., 2020; Trandafir and Cosmulescu, 2020; Shen et al., 2021; Medic et al., 2021/a; Medic et al., 2021/b; Li et al., 2023; Wu et al., 2023). Different papers reported quite similar quantities of phenolics in three different forms, however their sequence is always the same. The largest quantity of the phenolics is in free form, which rate is 51.1%-75.8%, followed by bound form (17.7%-38.0%) and esterified form (1.3%-18.7%) (Persic et al., 2018/a; Wu et al., 2021; Wang et al., 2022; Wu et al., 2023). The main source of the phenolic compounds (Colaric et al., 2005; Persic et al., 2018/a; Sheng et al., 2021; Medic et al., 2021/a; Jin et al., 2022) is pellicle, which contains almost 95% of the phenolic compounds, which can be detected in kernel (Slatnar et al., 2015). Positive and very strong correlations were found between the total phenolic content in free, esterified, and bound forms in pellicle and the pellicle color (0.920, 0.990, 0.940) (Wang et al., 2022). Kernels with a yellow pellicle contain more total phenolic content and flavonoids than a kernel with red pellicle (Trandafir et al., 2016/b; Peršić et al., 2018/b).

Beside the genetic backgrounds of the cultivars, just usage of the biostimulants is the only way to reach both aims described above, as they do not apply mechanical and chemical thinning in the nut tree production nowadays. The biostimulants have some positive effects on the plant characteristics, which can be seen from outside and can be measured inside. Usage of them is typical for herbaceous species mostly. In the nursery production usage of biostimulants (benzyladenine in 0.02%) increased feathering of the woody fruit bearing species such as apple, European plum, and cherries (Magyar and Hrotko, 2002). Furthermore, biostimulants had a positive influence on the growth of container grown shrubs, this application (applied biostimulants: Kelpak^®^ in 0.3%, Yeald Plus^®^ in 1.5%, Bistep in 0.5% dosages) improved their market value (Kovács et al., 2017). They increased the yield of different crops such as on faba bean (Lammas et al., 2022.), barley (Lakić et al., 2022), spring wheat (Lozowicka et al., 2022), wheat (Iwaniuk et al., 2022), sweet pepper (Ombódi and Toók, 2022), and potato (Mystkowska et al., 2022). Yield of peanuts was increased 25% to 296% after usage of the biostimulant treatments (Khudaykulov et al., 2021) compared to the control. Length of spike on spring barley plants increased significantly, and weight of grains of spring barley increased also up to 30% to 31% (Lammas et al., 2022) with 7% up to 14% on corn (Ajaj et al., 2020). Number of seeds per pods of faba bean become more with 22% (Lakić et al., 2022), oil content of peanut increased 2% to 7% after the application (Khudaykulov et al., 2021) compared to the control. Protein content of spring barley was increased by 11.9% to 12.8% by usage of Restart Zh (Lammas et al., 2022.). On ‘Oita 4’ mandarin easily extractable glomalin-related soil protein application combined with some other agricultural treatments (fruit bagging, reflective film mulching, and grass-proof cloth mulching) had positive effects on fruit quality (coloration value, hardness, fruit size, weight of pulp, peel and single fruit) (Lei et al., 2023). On strawberry, biostimulant applications had an effect on the yield and total anthocyanin content, but there were no effects on the total soluble sugar and acid content (Popovic et al., 2022). The biostimulant treatments decreased the mycotoxins contamination in the seeds of spring wheat (Lozowicka et al., 2022), and spring barley (Lammas et al., 2022). Among Eastern Bulgarian conditions ‘Lozen 1’ coriander contained from 2.9% to 9.6% more essential oil content after applying the biostimulant Fertigrain – 1.26% and the foliar fertilizer Masterblend – 1.25% (Georgieva et al., 2022). On oilseed plants usage of HL100, HLN55, and TH1-20% biostimulants decreased the saturated (by 3.5%, 1.74%, and 4.7%) and polyunsaturated fatty acids (by 2.74%, 0.59%, and 3.1%) content, however the monounsaturated fatty acid content increased (by 0.99%, 0.58%, 1,47%) (Petrova et al., 2023). Application of potassium (K) foliar fertilizers (such as Alga K Plus – 1%) had a moderate increase of cluster weight of wine grape cultivar ‘Zéta’. It also increased the sugar content in its juice and acceleration of ripening processes compared to the control vines. These results pointed out the positive effects of K foliar fertilization during the ripening process due to K deficiency of grapevines (Zsigrai and Juhász, 2000).

Currently, there is no data about effects of biostimulants’ applications on walnut, therefore the possible responses of this nut crop should be checked. Aim of this study was to examine the responses of nut size characters and some phenolic compounds of walnut kernel on usage of different biostimulants applied in a bearing orchard.

## 2 Materials and methods

### 2.1 Plant material and orchard system

The trial was conducted in a commercial orchard of Hilltop Vinery Ltd. located in Kocs (North-West Hungary, GPS coordinates 47°34’57” N, 18°15’04” E, 193 m above sea level). The orchard was planted in fall of 1999 with grafted trees. The ‘Alsószentiváni 117’ scion was grafted on *Juglans regia* selected seedling rootstocks, and planted out 10 x 10 m in the rows and between the rows, and trained to a central leader canopy. The orchard was not irrigated. The observed trees produced 30 to 50 cm one-year-old shoots during the previous year, which means medium condition in the practice.

The examined cultivar, derived from the walnut breeding program of the Fruit Research Centre of the Hungarian University of Agriculture and Life Sciences in Budapest – Érd (Hungary), is a selection from the local population. It has early budburst (early April) and the earliest harvest time (second decade of September) in the Hungarian walnut assortment. It is a proterandric cultivar. It has an oval, large nut size, which means 33 to 35 mm in diameter, large kernel recovery (40 to 45%), and a light brown shell and kernel color (Bujdosó et al., 2022).

### 2.2 Soil and climatic conditions

The trial was planted on loamy soil with high lime (pH = 7.5, total lime content in the top 120 cm layer 5%) and humus content (0,8–1.5%). Considering the Arany-type cohesion index (Dobos et al., 2010) the K_A_ = 49 refers to medium compactness. Meteorological conditions of the site are presented in **Table 1**. The 2020 spring was warmer during the pistillate flowering receptivity, than spring of 2021.

**Table 1:** Meteorological data during the data collection in 2020 and 2021.

Parameters	2020	2021
average yearly temperature	11.4°C	10.6°C
average yearly temperature during the growing season (March–September)	16.1°C	15.4°C
number of frosty nights during spring (between March and May)	28	34
average air temperature during spring (between March and May)	2.5°C	2.9°C
average air temperature during the pistillate flowering receptivity	11.6°C	9.6°C
days with frosty nights during the pistillate flowering receptivity	1 day	2 days
average yearly luminous flux	1015 l/m^2^/day	996 l/m^2^/day
annual average of sunshine hours	2065	2001
average yearly precipitation	434.1 mm	404,5 mm

### 2.3 Treatments

The biostimulants used in the trial were tested in a small plot trial design. 10-10 sample walnut trees per treatment were selected in such a way as to represent the different growing conditions of the area. Five replications of the samples taken per each treatment and the untreated control, where one sample consisted of 50 nuts, thus the physical parameters of 250 nuts per treatment were measured individually. Three biostimulants were tested that can also be used in organic farming to increase yields and improve nut quality: Wuxal Ascofol (Kwizda Agro Hungary Kft.), Alga K Plus (Leili Agrochemistry Co. LTD) and Kondisol (Huminisz Kft). Each tested product was applied according to the product description and instructions.

*Used biostimulants:*

*Wuxal Ascofol:* This biostimulant is from algae extracts derived from seaweed *Ascophyllum nodosum*, which belongs to the group of yellow algae. Its active ingredients are N – 30 g/l; K – 20 g/l; B – 38 g/l; Mn – 10 g/l; Zn – 6,3 g/l. In the case of stone fruit species, the recommended dose to increase the size of the fruit (to stimulate cell division and elongation) is 3 l/ha. The application was foliar spraying after leafing, when the leaf surface was sufficiently developed. Due to its natural plant hormone content, it increases the stress tolerance of plants, especially in the early phase of the fruit growth, and stimulates cell division and elongation, which can be expected to improve the quality and quantity of the crop. Due to its high microelement content, it promotes fruit setting and strengthens the plants’ natural resistance and improving frost tolerance (Website 1).

*Alga K Plus:* It is a foliage fertilizer with a very high potassium (K_2_O 30%) and algae content. The combination of the algae extract and potassium found in it can be very effective in the case of walnuts, especially as walnuts are a potassium-demanding plant. Its application is recommended for most of fruit species from the beginning of ripening. The combination of the algae extract and potassium effectively improves the fruit quality, especially the sugar and dry matter content, the amount of flavoring substances, and contributes to the development of the marketable color. To improve the quality of grape and fruit crops, to increase the sugar content, to add color, the advised dose is 3–5 kg/ha and the maximum concentration is 0.7–1.0%. The dose of the Alga K Plus treatment in walnuts was 3 kg/ha (Website 2).

*Kondisol*: The physiological effect of the Kondisol product family is primarily due to different sized humic acids and fulvic acids. In addition, the products contain enzymes, co-enzymes, polysaccharides, various macro-, micro- and meso-elements. Mechanism of action of the product is humic acids have a “carrier” role, since they significantly contribute to the faster uptake and better utilization of macro-, micro- and meso-elements, as they adsorb metal ions in the form of metal humates. Due to the oxygen-carrying and respiratory processes-accelerating effect of humic acids (they stimulate peroxidase activity), Kondisol enhances root formation, plant growth, enzyme activity and protein synthesis resulting to increased yields. It can be used to reduce the year effect, to treat and eliminate relative nutrient deficiencies (ion antagonisms: e.g. P-Zn, K-Mg). In the case of unfavorable soil conditions (e.g. compaction, excessive water saturation) it helps the uptake and utilization of mineral elements and nutrients. It also reduces the scale of stress on plants in extreme weather and stress situations (e.g. drought, cold weather, heavy rainfall). It is characterized by fast uptake and excellent utilization (several times faster related to traditional foliar fertilizers). Applied dose in the treatments was 6 l/ha, which matched the application recommendations (Website 3).

**Timing of treatments:**

The products presented above were applied with a mist blower motorized spraying machine to evenly apply the treatment on the canopy. Date of application for all three products was on 11^th^ May 2020 and on 7^th^ May 2021. The different treatments were separated from each other by a safety (untreated) row of walnut trees.

**Sample collection:**

Nut samples from three treatments and the untreated control for the laboratory tests were collected during the harvest time (when 50% of the green husks were opened). A total of 250 nuts per treatment (in 5 replications, 50 nuts per replication) were taken randomly. The sample collection date for Alga K Plus and Kondisol was on 24^th^ September 2020. At this time, the nuts of the trees treated with Wuxal Ascofol were still immature (their green husks did not crack). In the case, the samples were collected one week later, on 30^th^ September 2020. In 2021, all samples were collected on 1^st^ October.

During laboratory observations the phenolic compounds (pirocatechin, catechin, chlorogenic acid, rutin, quercetin, juglon, cinnamic acid, and gallic acid) and their physical parameters (nut size (nut length, nut diameter), dried nut weight, dried kernel weight, kernel recovery (dried kernel weight/dried nut weight)) were checked.

### 2.4 Chemicals, sample preparation, analytical conditions

#### 2.4.1 Chemicals for sample preparation

Analytical HPLC grade standards of different phenolic compounds such as catechin (PubChem CID: 73160), chlorogenic acid (PubChem CID: 1794427), cinnamic acid (PubChem CID: 44539), epicatechin (PubChem CID: 72276), gallic acid (PubChem CID: 370), juglone (PubChem CID: 3806), pyrocatechin (PubChem CID: 161125), quercetin (PubChem CID: 5280343), rutin (PubChem CID: 5280805) and the solvents phosphoric acid and methanol (MeOH), were purchased from Sigma Aldrich Chemical Co. (St. Louis, MO, USA). The standards (0.5 g mL-1) were dissolved in methanol and a 100× dilution was used as the working standard for HPLC.

#### 2.4.2 Sample preparation

1000 g of dried kernels –contained their seed coats– (drying at 30°C up to 10% of moisture content) were examined. 2 g samples were taken to Falcone-tubes and extracted in 10 mL methanol for 12 hours in the dark (4°C, using an Edmund Bühler SM 30 control shaker on 250 rpm min^-1^). The supernatant was decanted and centrifuged in Eppendorf tubes in a Hettich Mikro 22R (Andreas Hettich GmbH & Co. KG Tuttlingen, Germany) centrifuge (15000 rpm min^-1^ for 5 min). The supernatant was filtered on a 0.45 μm MILLEX^®^ HV Syringe Driven Filter Unit (SLHV 013 NL, PVDF Durapore), purchased from Millipore Co. (Bedford, MA, USA), and injected into the HPLC system. The quantities of the individual phenolic compounds are given in mg g^-1^.

#### 2.4.3 Analytical conditions

The analysis carried out at the HPLC Laboratory of Department of Fruit Growing, Institute of Horticultural Science, Hungarian University of Life Sciences. The WATERS High Performance Liquid Chromatograph (purchased from Waters Co., Milford, MA, USA) was equipped with 2487 Dual λ Absorbance Detector, a 1525 binary HPLC pump, and in-line degasser, a column thermostat (set at 35°C) and an 717^plus^ auto sampler (set at 5°C) and was controlled using EMPOWER TM^2^ software. An Atlantis dC18 5 μm 4.6X150 mm column (Waters Co., Milford, MA, USA) was installed. The gradient mobile phase was A: H_2_O:MeOH:H_3_PO_4 = _940:50:1; B: MeOH (0–30 min: A 100%–10%, 30–30.1 min: 10%–100%, 30.1–31: A 100%) with a flow rate 1 cm^3^ min^-1^, the pressure in the column was 2500 ± 10 psi at a column temperature of 30°C. The running time was 35 minutes. Each injected volume was 20 μL. The sampling rate was 10 pt sec^-1^, and the phenolic components were monitored at a wavelength of 280 nm.

### 2.5 Statistical evaluation

The data derived from compositional analyses were evaluated using the SPSS software (IBM SPSS 27.0, Chicago, IL, USA). The letters, a, b, c indicate significantly different groups at SD_5%_, while cultivars that are not significantly different are indicated with the same letter. Values represent the mean and standard deviation of five replicates from each sample.

## 3 Results

**Figure 1:**
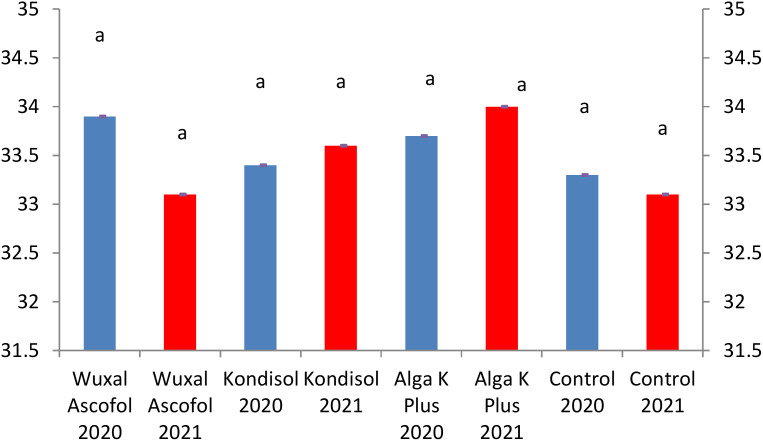
Effects of different biostimulants on nut length (mm) of ‘Alsószentiváni 117’ walnut cultivar (SD_5%(2020)_=0.6, SD_5%(2021)_=0.6).

The applied biostimulants had different effects on the physical parameters of the nuts. In the case of nut length, all treatments had positive effects compared to the control during both years (**Figure 1**).

**Figure 2:**
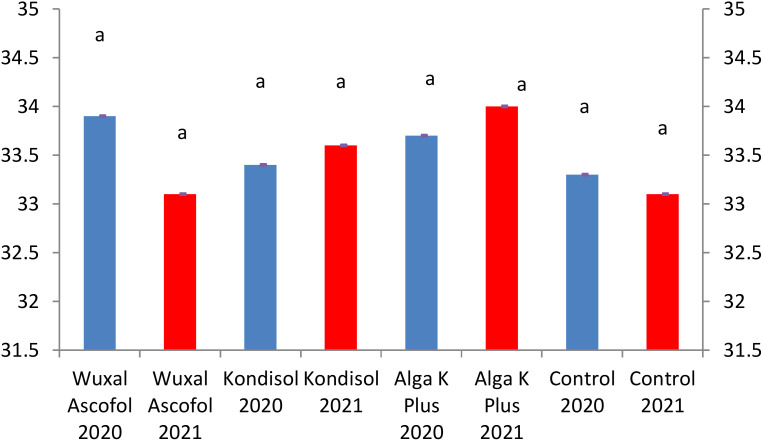
Effects of different biostimulants on nut diameter (mm) of ‘Alsószentiváni 117’ walnut cultivar (SD_5%(2020)_=0.4, SD_5%(2021)_=0.25).

All treatments resulted to a larger nut diameter than the control. In both years, the diameter of the examined nuts reached the lowest border of first grade category (**Figure 2**).

**Figure 3:**
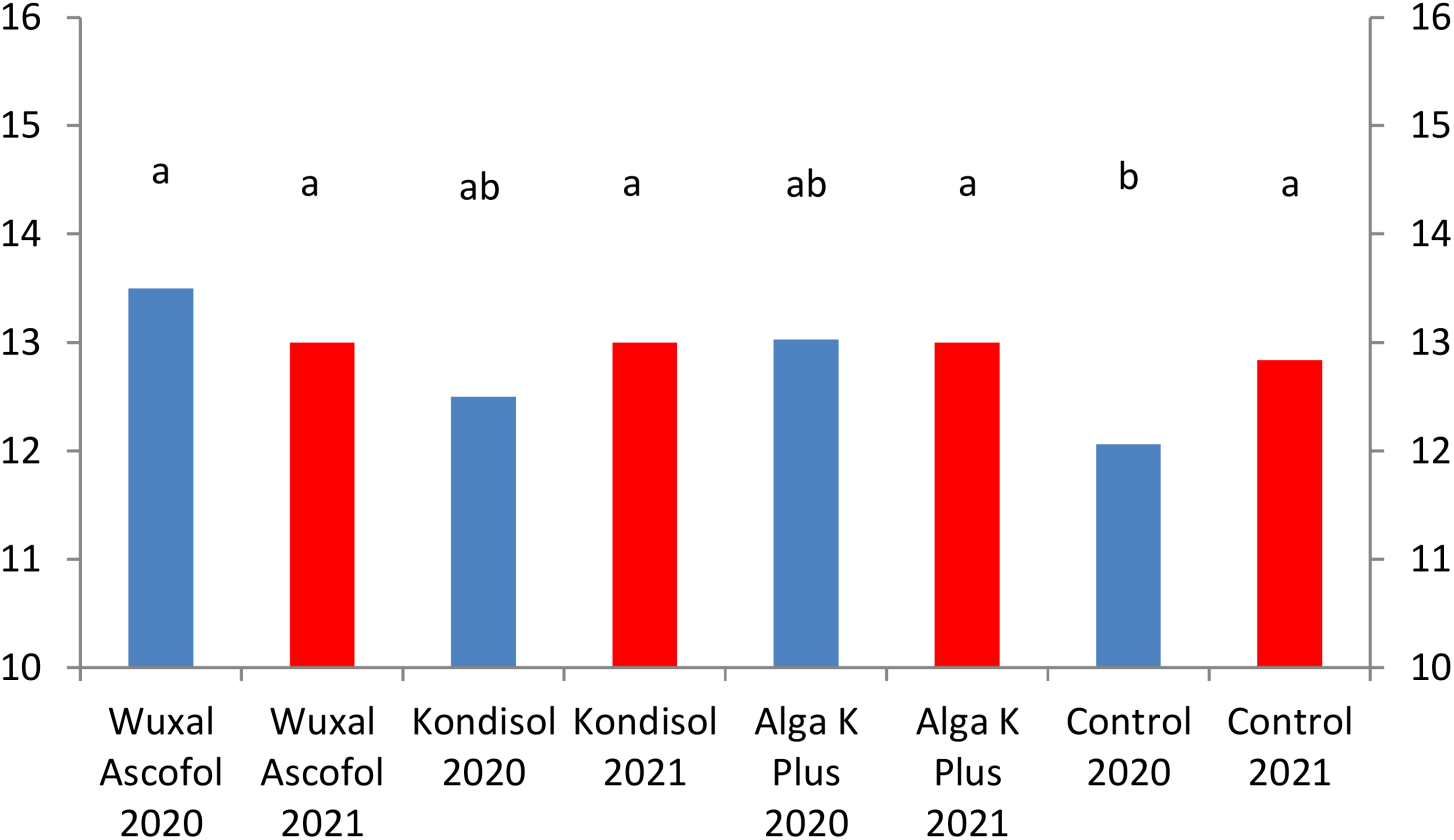
Effects of different biostimulants on dried nut weight (g) of ‘Alsószentiváni 117’ walnut cultivar (SD_5%(2020)_=0.5, SD_5%(2021)_=0.3).

In both observed years, all treatments resulted to heavier dried nut weight compared to control. Among the treatments, the heaviest dried nut weight was produced by Wuxal Ascofol in 2020. In 2021, nuts after all treatments had the same value of dried nut weight. The Alga K Plus treatments reached the same values in both observed years; there was no effect of year in this case (**Figure 3**).

**Figure 4:**
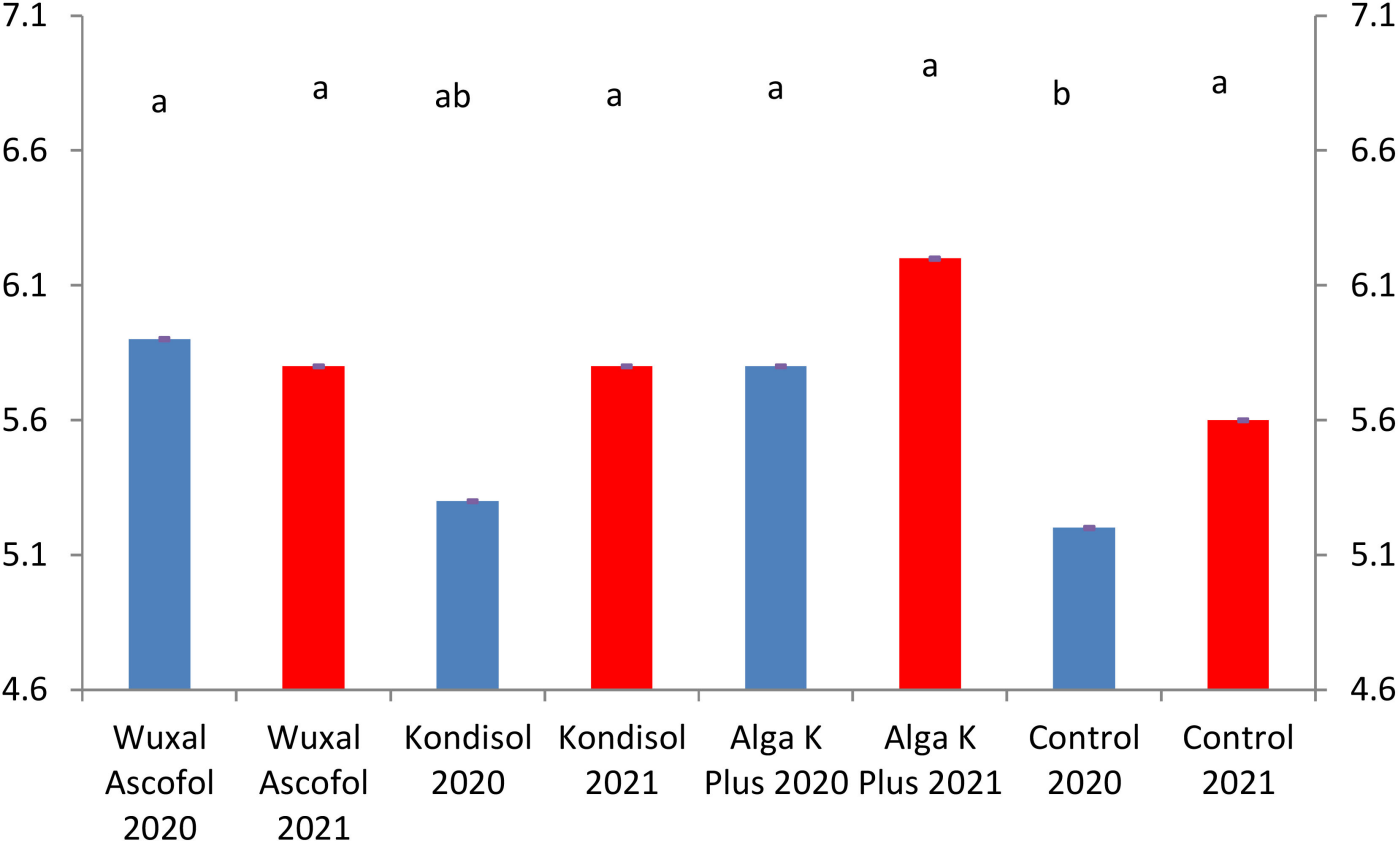
Effects of different biostimulants on dried kernel weight (g) of ‘Alsószentiváni 117’ walnut cultivar (SD_5%(2020)_=0.4, SD_5%(2021)_=0.5).

All treated kernels were heavier than the control in 2020 and in 2021. Among the Wuxal Ascofol treatments, a heavier kernel weight was produced in 2020, than in 2021. Among the Kondisol, Alga K Plus, and control treatments, heavier dried kernel weights were measured in 2021 compared to 2020 (**Figure 4**).

**Figure 5:**
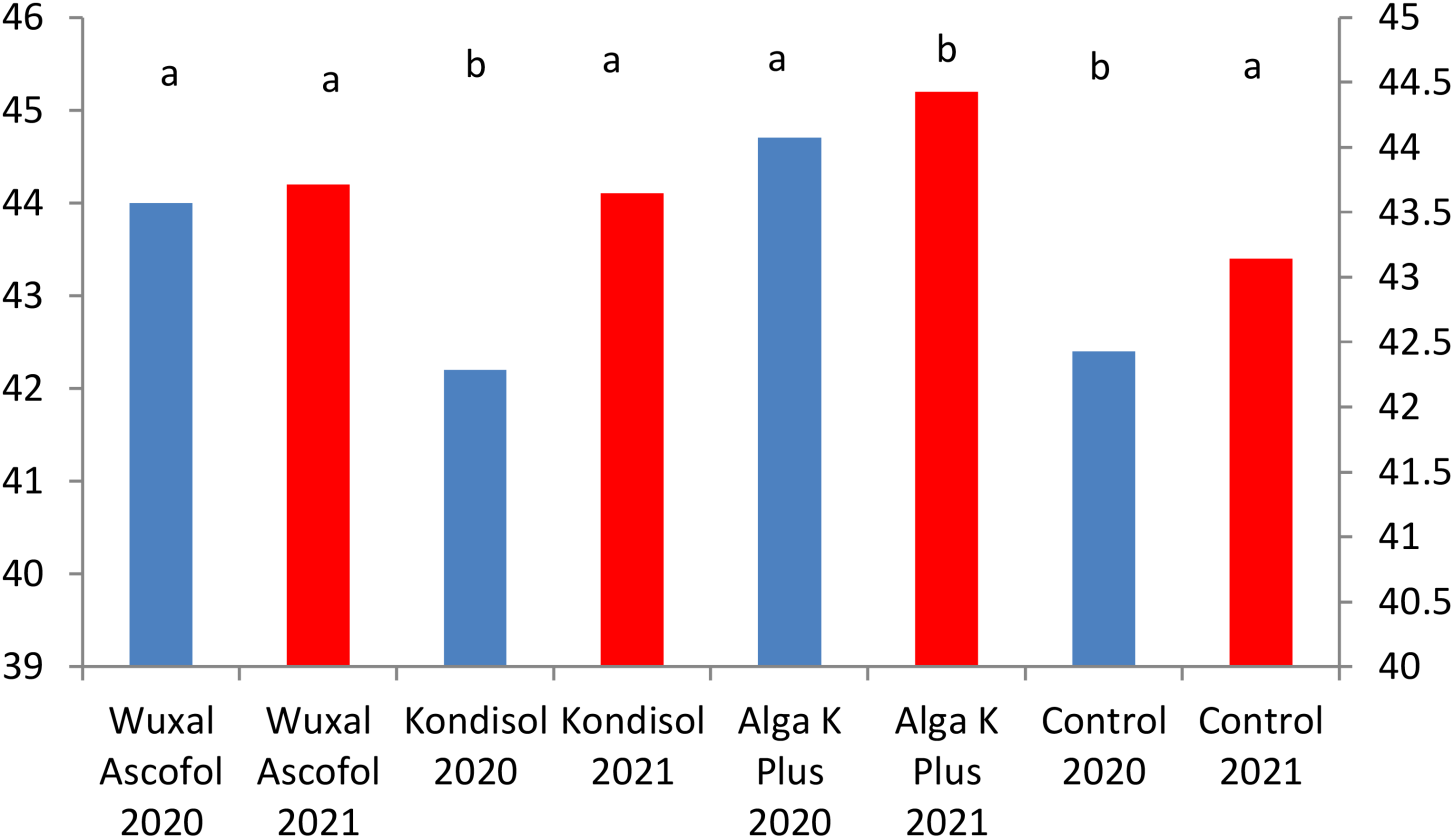
Effects of different biostimulants on kernel recovery (%) of ‘Alsószentiváni 117’ walnut cultivar (SD_5%(2020)_=6.1, SD_5%(2021)_=5.2).

The applied biostimulant treatments had positive effects on the kernel recovey in both observed years, except the Kondisol treatment had a significantly negative effect in 2020 compared to the other treatments (**Figure 5**).

There were big differences in the quantity of phenolic compounds detected in the kernel between the two years during our research. In 2021, all observed compounds had higher concentrations compared to their quantities measured in 2020.

**Figure 6:**
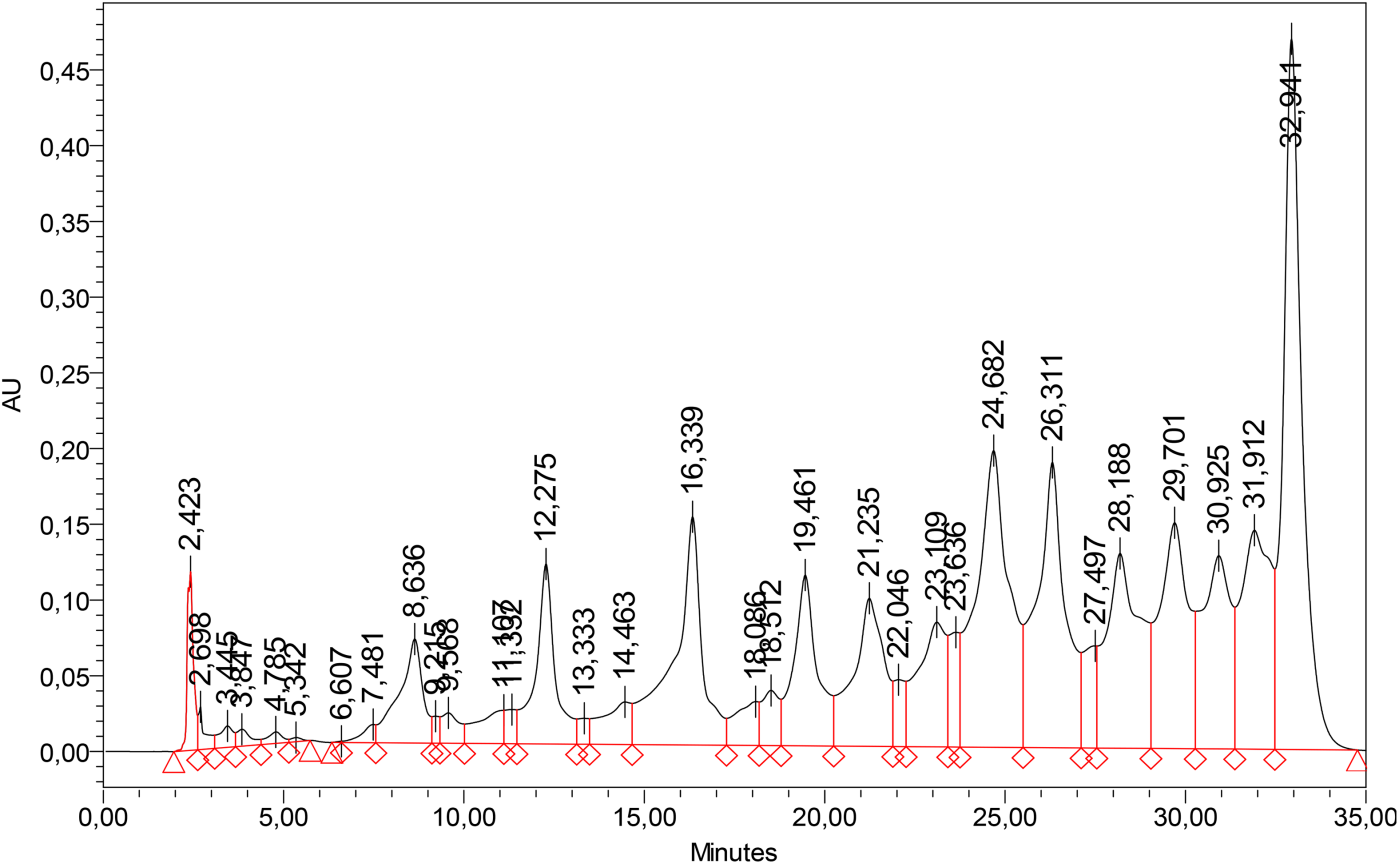
Typical HPLC chromatogram of walnut extraction (‘Alsószentiváni 117’) at 280 nm.

**Figure 6** shows a typical HPLC chromatogram of ‘Alsószentiváni 117’ (retention times of observed phenolic compounds; pirocathecin 8.6 min, catechin 9,5 min, chologenic acid 12,2 min, rutin 16.3 min, quercetin 18.5 min, juglon 19.4 min, cinnamic acid 21.2 min, gallic acid 32.9 min).

**Figure 7:**
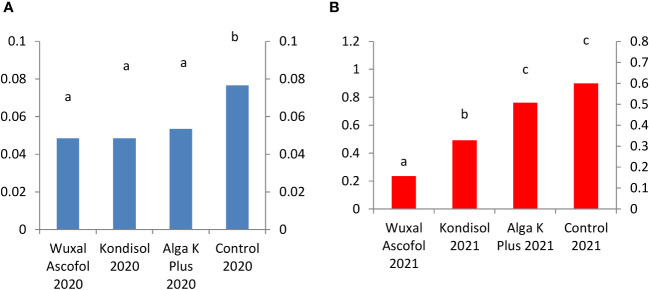
Effects of different biostimulants on pirocatechin content (mg/g kernel) of ‘Alsószentiváni 117’ walnut kernel in 2020 **(A)** and 2021 **(B)**(SD_5%(2020)_=0.01 SD_5%(2021)_=0.15) The scale on the Y axis is different on the a and b figures due to quantities caused by effects of the year.

In 2021, the pirocatechin concentration was 5 to 14-fold higher than in 2020. In 2020, the highest concentration of pirocatechin was measured in control, followed kernels treated with Alga K Plus, Wuxal Ascofol, and Kondisol. In 2021, the quantity of this compound decreased in all kernels with applied biostimulants treatments compared to the control (**Figure 7**).

**Figure 8:**
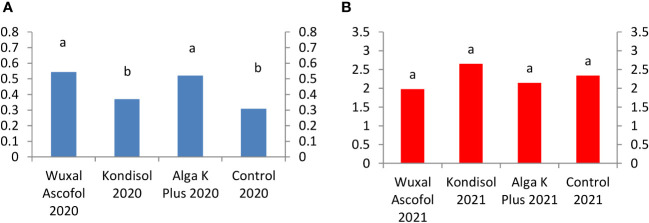
Effects of different biostimulants on catechin content (mg/g kernel) of ‘Alsószentiváni 117’ walnut kernel in 2020 **(A)** and 2021 **(B)** (SD_5%(2020)_=0.05, SD_5%(2021)_=0.6) The scale on the Y axis is different on the a and b figures due to quantities caused by effects of the year.

For catechin, kernels from trees treated with Wuxal Ascofol and Alga K Plus reached the highest concentration in 2020. In the case of 2021, the Kondisol treatment had the highest concentration followed by control, Alga K Plus, and Wuxal Ascofol treatments. The concentration of catechin was 7-fold higher in the control and Kondisol treatment, followed by a 4-fold after Wuxal Ascofol and Alga K Plus treatments in 2021 compared to 2020 (**Figure 8**).

**Figure 9:**
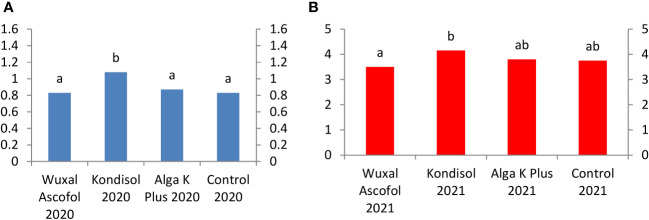
Effects of different biostimulants on chlorogenic acid content (mg/g kernel) of ‘Alsószentiváni 117’ walnut kernel in 2020 **(A)** and 2021 **(B)** (SD_5%(2020)_=0.11, SD_5%(2021)_=0.3) The scale on the Y axis is different on the a and b figures due to quantities caused by effects of the year.

The sequence of chlorogenic acid content was similar in both observed years, but the chlorogenic acid concentration was 4-fold higher after all treatment applications in 2021, than in 2020. Kernels treated with Kondisol reached the highest concentration followed by Alga K Plus, as well as Wuxal Ascofol treatments and control (**Figure 9**).

**Figure 10:**
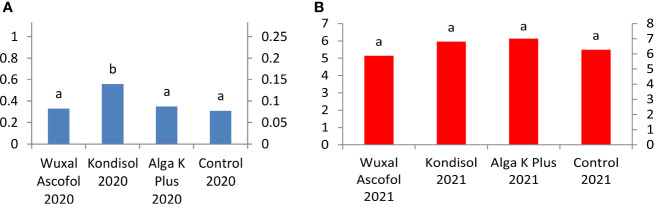
Effects of different biostimulants on rutin content (mg/g kernel) of ‘Alsószentiváni 117’ walnut kernel in 2020 **(A)** and 2021 **(B)** (SD_5%(2020)_=0.12, SD_5%(2021)_=0.3) The scale on the Y axis is different on the a and b figures due to quantities caused by effects of the year.

There were large differences in the rutin concentration between 2020 and 2021. Kernels from Alga K Plus treatment and the control produced 17-fold, Wuxal Ascofol-treatment 15-fold, and Kondisol treatment had 10-fold higher rutin quantity in 2021 than in 2020. Among the biostimulants the sequence was different in both observed years. In 2020, kernels with Kondisol had the highest rutin concentration, followed by the two other biostimulants and control, which reached the same amount. In 2021, kernels with Alga K Plus application produced the highest concentration of rutin, followed by Kondisol, control, and Wuxal Ascofol treatments (**Figure 10**).

**Figure 11:**
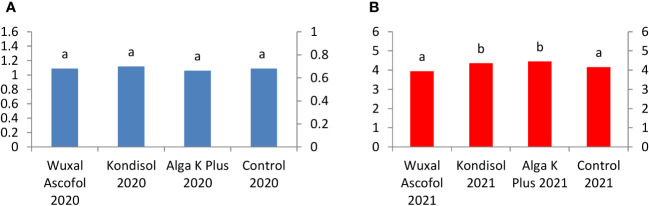
Effects of different biostimulants on quercetin content (mg/g kernel) of ‘Alsószentiváni 117’ walnut kernel in 2020 **(A)** and 2021 **(B)** (SD_5%(2020)_=0.08, SD_5%(2021)_=0.22) The scale on the Y axis is different on the a and b figures due to quantities caused by effects of the year.

The quercetin concentration increased 4-fold from 2020 to 2021 after all treatments. Within the observed years, there were quite similar values. In 2020, the Kondisol treatment reached the highest value, followed by control, Wuxal Ascofol, and Alga K Plus applications. In 2021, Alga K Plus, Kondisol treatments, control, and Wuxal Ascofol applications were in descending order (**Figure 11**).

**Figure 12:**
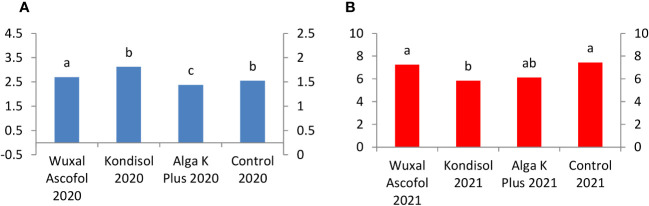
Effects of different biostimulants on juglon content (mg/g kernel) of ‘Alsószentiváni 117’ walnut kernel in 2020 **(A)** and 2021 **(B)** (SD_5%(2020)_=0.2, SD_5%(2021)_=1.1) The scale on the Y axis is different on the a and b figures due to quantities caused by effects of the year.

Juglon is one of the most important compounds of walnut, therefore we put more attention on it. The control, kernels with Wuxal Ascofol, and Alga K Plus treatments produced 3-fold, the kernels with Kondisol reached a 2-fold higher juglon concentration in 2021 than in 2020. Among the treatments the sequence of juglon concentration was different between 2020 and 2021. In 2020, Kondisol, Wuxal Ascofol, control, and Alga K Plus treatments were the decreasing order. However, the control reached the highest concentration of juglon in 2021, followed by Wuxal Ascofol, Alga K Plus, and Kondisol applications (**Figure 12**).

**Figure 13:**
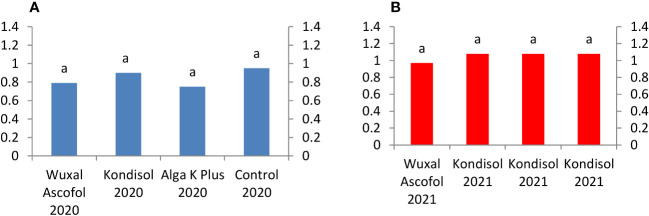
Effects of different biostimulants on cinnamic acid content (mg/g kernel) of ‘Alsószentiváni 117’ walnut kernel in 2020 **(A)** and 2021 **(B)** (SD_5%(2020)_=0.9, SD_5%(2021)_=0.7) The scale on the Y axis is different on the a and b figures due to quantities caused by effects of the year.

The cinnamic acid concentration showed a 1.2 to 1.4-fold increase from 2020 to 2021. During 2020, the control produced the highest cinnamic acid quantity followed by samples with Kondisol, Wuxal Ascofol, and Alga K Plus treatments. In 2021, again the control reached the highest cinnamic acid concentration followed by Kondisol, Alga K Plus, and Wuxal Ascofol treatments (**Figure 13**).

**Figure 14:**
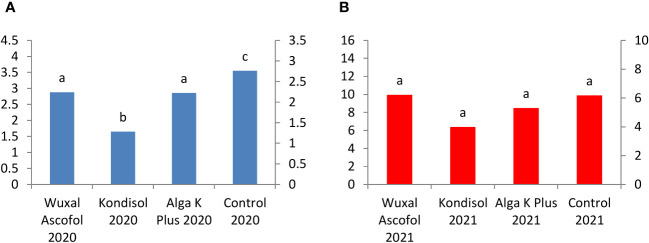
Effects of different biostimulants on gallic acid content (mg/g kernel) of ‘Alsószentiváni 117’ walnut kernel in 2020 **(A)** and 2021 **(B)** (SD_5%(2020)_=0.5, SD_5%(2021)_=1.8) The scale on the Y axis is different on the a and b figures due to quantities caused by effects of the year.

In 2021, we detected 3 to 4-fold higher gallic acid concentration after all treatments than in 2020. In 2020, the control had the highest gallic acid quantity, followed by samples treated with Wuxal Ascofol, Alga K Plus, and Kondisol. In the next year, we measured the highest concentration in the control and in a sample with Wuxal Ascofol, Alga K Plus and Kondisol treatments (**Figure 14**).

## 4 Discussions

The applied biostimulants had different effects on nut and kernel characteristics as well as the quantity of phenolic compounds in the kernels during the two trial years. All nut size and kernel traits increased after the biostimulants treatments in both observed years, except in the case of kernel recovery after Kondisol treatment in 2020. There was a significant difference between the treated nut length and the control in 2020, this difference was not observed in 2021. There were no significant differences in the nut diameter during the observed two years. There were significant difference in dried nut weight between the control and nuts treated with Wuxal Ascofol in 2020. There were significant difference in dried kernel weight between the control and nuts treated with Wuxal Ascofol in 2020. In 2021, the Alga K Plus produced a significantly positive effect on kernel recovery than the other treatments and control. It is clear, that there were huge differences between years of 2020 and 2021. The early spring weather during the pistillate flowering receptivity was warmer in 2020 than in 2021, which might have caused this difference.

According to Wielgolaski (2001) climatic factors correlated well to the phenological stages, and the air temperature is reported to be the major force for the onset of the early spring phenological stages (Rodriguez-Rajo et al., 2003; Wielgolaski, 2003; Crepinsek et al., 2009; Cosmulescu et al., 2010; Crepinsek et al., 2012; Bîrsanu Ionescu and Cosmulescu et al., 2017; Cosmulescu et al., 2015; Bujdosó et al., 2022) such as pistillate flower receptivity. Negative relationships between the heat demand of early spring phenological stages and daily air temperatures indicate that warm weather is required for early flowering (Emberlin et al., 2007). ‘Alsószentiváni 117’ has one of the earliest pistillate flowering receptivity listed on the Hungarian walnut assortment.

Respect to phenolic content, this study analyzed eight compounds. In 2020, pirocatechin content was significantly lower than the control. In 2021, again the control had significantly higher pirocatechin content, than those treated with Wuxal Ascofol and Kondisol. Catechin content of kernels treated with Wuxal Ascofol and Alga K Plus were significantly higher than in control and Kondisol in 2020. In the next year, there was no significant difference in the catechin content in all treated kernels. The chlorogenic acid content was higher in kernels treated with Kondisol in both years. In 2020, the Kondisol treatment had significant higher value in chologenic acid content. In 2021, the difference in chologenic acid content was only significant between kernels treated with Wuxal Ascofol and Kondisol. In rutin content, significant differences were only observed in kernels treated with Kondisol in 2020. For the case of quercetin content, kernels treated with Kondisol and Alga K Plus had the highest quantity. They were significantly different from those treated with Wuxal Ascofol and the control in 2021. Significant difference in juglon content was observed between kernels treated with Alga K Plus and Wuxal Ascofol in 2020. In the next year, the untreated kernels had the highest juglon content, followed by kernels treated by Wuxal Ascofol, both were significant different from kernels treated with Kondisol. There was no significant difference in the cinnamic acid content in both observed years. In the case of gallic acid, untreated kernels had the highest content, which were significantly different from all treated kernels in 2020. There was no significant difference in gallic acid in 2021.

Our results confirm statements of Popovic et al., 2022, and Petrova et al., 2023 that different effects of the biostimulants on the compounds can be detected.

## 5 Conclusions

The biostimulants affect the plants at different levels, and we have to underline that responses of woody fruit species on biostimulants depend not only on biostimulant – host plant interactions, but also on weather conditions during and after their application. When warm spring weather conditions occurred, not only significant differences in the nut size characteristics were observed, but all examined characters increased.

## Data availability statement

The raw data supporting the conclusions of this article will be made available by the authors, without undue reservation.

## Author contributions

GS: Conceptualization, Data curation, Formal Analysis, Methodology, Supervision, Writing – original draft, Writing – review & editing. GB: Supervision, Validation, Writing – original draft, Writing – review & editing. MC: Formal Analysis, Writing – original draft. OT: Data curation, Formal Analysis, Writing – original draft. LK: Data curation, Supervision, Writing – original draft. RO: Data curation, Formal Analysis, Writing – original draft. GF: Data curation, Formal Analysis, Methodology, Supervision, Validation, Writing – original draft, Writing – review & editing. GV: Conceptualization, Data curation, Formal Analysis, Methodology, Supervision, Validation, Writing – original draft, Writing – review & editing.

## References

[B1] Ajaj H. A. Abood N. M. Noiman Z. A. J. Sirhan S. A. (2020). Response of several maize (Zea mays L.) cultivars to spray with Novalon biostimulant. Mesopotamia J. Agriculture. 47, 407–427.

[B46] Akca Y. Sutyemez M. Yilmaz S. Karadag H (2016). Book of proceedings. “The new walnut variety breeding program in Turkey,” in Abstract received from the AGROSYM 2016 Conference Universty of East Sarajevo, Jahorina, Bosnia and Herzegovina. doi: 10.7251/AGRENG1607064

[B2] Bîrsanu Ionescu M. Cosmulescu S. (2017). Effect of climatic conditions onflowering of walnut genotypes in Romania. J. Nuts. 8 (2), 161–167.

[B4] Bujdosó G. Lengyel-Kónya É. Berki M. Végh A. Fodor A. Adányi N. (2022). Effects of phenolic compounds on walnut bacterial blight in the green husk of hungarian-bred cultivars. Plants 11, 2996. doi: 10.3390/plants11212996 36365449 PMC9657124

[B3] Bujdosó G. Végvári G.Y. Hajnal V. Ficzek G. Tóth. M. (2014). Phenolic profile of the kernel of selected Persian walnut (*Juglans regia* L.) cultivars. Not. Bot. Horti. Agrobo. 1 (42), 24–29. doi: 10.15835/nbha4219400

[B5] Colaric M. Veberic R. Solar A. Hudina M. Stampar F. (2005). Phenolic acids, syringaldehyde and juglone in fruits of different cultivars of Juglans regia L. J. Agr. Food. Chem. 53 (16), 6390–6396. doi: 10.1021/jf050721n 16076123

[B6] Cosmulescu S. Baciu A. Botu M. Achim G. (2010). Environmental factors'influence on walnut flowering. Acta Hortic. 861, 83–88. doi: 10.17660/ActaHortic.2010.861.10

[B7] Cosmulescu S. Baciu A. Gruia M. (2015). Influence of climatic factors on the phenology spring in southern Oltenia (Romania). J. Horticult. Forest. Biotechnol. 19 (1), 147–157.

[B8] Crepinsek Z. Solar M. Stampar F. Solar A. (2009). Shifts in walnut (Juglans regia L.) phenology due to increasing temperatures in Slovenia. J. Hortic. Sci. Biotechnol. 84 (1), 59–64.

[B9] Crepinsek Z. Stampar F. Kajfez-Bogataj L. Solar A. (2012). The response of Corylus avellana L. phenology to rising temperature in north-eastern Slovenia. Int. J. Biometeorol. 56 (4), 681–694. 21786017 10.1007/s00484-011-0469-7

[B10] Dobos E. Bialko T. Micheli E. Kobra J. (2010). ““Legacy soil data harmonization and database development”,” in Digital Soil Mapping, Progress in Soil Science. Eds. Boettinger J. L. Howell D. W. Moore A. C. Hartemink A. E. Kienast-Brown S. (Dordrecht, Germany: Springer), 215–223.

[B11] Emberlin J. Smith M. Close R. Adams-Groom B. (2007). Changes in the pollen season of the early flowering trees Alnus spp. and Corylus spp. in Worcester, United Kingdom 1996–2005. Int. J. Biometeorol. 51, 181–191. doi: 10.1007/s00484-006-0059-2 17024396

[B12] FAO (2023) Database. Available at: https://www.fao.org/faostat/en/#data/QCL (Accessed 1st June 2023).

[B13] Georgieva R. Delibaltova V. Chavdarov P. (2022). Change in agronomic characteristics and essential oil composition of coriander after application of foliar fertilizers and biostimulants. Ind. Crop Prod. 181, 114819. doi: 10.1016/j.indcrop.2022.114819

[B14] Gharibzahedi S. Mousavi M. Hamedi M. Khodaiyan F. (2014). Determination and characterization of kernel biochemical composition and functional compounds of Persian walnut oil. J. Food. Sci. Technology-mysore. 51, 1–9. doi: 10.1007/s13197-011-0481-2 PMC3857422 24426045

[B15] Iordănescu O. Radulov I. Buhan I. Cocan I. Berbecea A. Popescu I.. (2021). Physical, nutritional and functional properties of walnuts genotypes (Juglans regia L.) from Romania. Agronomy 11, 1092. doi: 10.3390/agronomy11061092

[B16] Iwaniuk P. Konecki R. Kaczyński P. Rysbekova A. Lozowicka B. (2022). Influence of seven levels of chemical/biostimulant protection on amino acid profile and yield traits in wheat. Crop J. 10, 4. doi: 10.10.1016/j.cj.2021.12.007

[B17] Jin F. Wang Y. Huang R. Li B. Zhou Y. Pei D. (2022). Phenolic extracts from colored-walnut pellicles: antioxidant efficiency in walnut oil preservation. Int. J. Food Prop. 25, 1458–1471. doi: 10.1080/10942912.2022.2082466

[B18] Kafkas E. Attar S. Gundesli M. Özcan A. Ergun M. (2020). EMMFT-2020. Phenolic and fatty acid profile, and protein content of different walnut cultivars and genotypes ( Juglans regia L.) grown in the USA. Int. J. Fruit Science. 20, 1711–1720. doi: 10.1080/15538362.2020.1830014

[B19] Khudaykulov J. Togaeva S. Kashkabaeva C. Abirov Z. Shodmonov S. (2021). “Effects of terms and norms of biostimulant application on local peanut yield and seed quality,” in E3S Web of Conferences EDP Sciences: Les Ulis, France. 244. doi: 10.1051/e3sconf/202124402046

[B21] Kónya É. Bujdosó G. Berki M. Nagy-Gasztonyi M. Adányi N. (2015). Effect of short term storage on walnut fruit quality. Acta Aliment. Hung. 4 (44), 601–608. doi: 10.1556/066.2015.44.0033

[B20] Kovács D. Magyar L. Sütöriné D. M. Hrotkó K. (2017). Treatments affecting the growth of Forsythia x intermedia Zabel. ‘Beatrix Farrand’ container grown shurbs. Gradus 4 (2), 284–289.

[B22] Lakić Ž. Nozinic M. Antic M. Popovic V. (2022). The influence of the biostimulant on the yield components and yield of faba bean (Vicia faba var. minor). Not. Bot. Horti Agrobo. 50, 12998. doi: 10.15835/nbha50312998

[B23] Lammas M. Shitikova A. Savoskina O. (2022). The role of growth biostimulants in obtaining a high-quality crop of spring barley plants. АгроЭкоИнфо 6, 7–7. doi: 10.51419/202126607

[B24] Lei A.-Q. Yang Q.-H. Zhang Y. Liao W.-Y. Xie Y.-C. Srivastava A.. (2023). Agronomic practices alter regulated effects of easily extractable glomalin-related soil protein on fruit quality and soil properties of satsuma mandarin. Agronomy 13, 881. doi: 10.3390/agronomy13030881

[B25] Li M. Lu P. Wu H. Souza T. Suleria H. (2023). *In vitro* digestion and colonic fermentation of phenolic compounds and their bioaccessibility from raw and roasted nut kernels. Food Funct. 14, 2727–2739. doi: 10.1039/d2fo03392e 36852611

[B26] Lozowicka B. Iwaniuk P. Konecki R. Kaczyński P. Kuldybayev N. Dutbayev Y. (2022). Impact of diversified chemical and biostimulant protection on yield, health status, mycotoxin level and economic profitability in spring wheat (Triticum aestivum L.) cultivation. Agronomy 12, 258. doi: 10.3390/agronomy12020258

[B27] Magyar L. Hrotko K. (2002). Effect of 6-benzyladenine (BA) and gibberellic acid (GA4+7) application on feathering of plum cultivars in nursery. Acta Hortic. 577, 345–349. doi: 10.17660/ActaHortic.2002.577.59

[B29] Medic A. Hudina M. Jakopič J. Solar A. Veberic R. (2021/b). Identification and quantification of the major phenolic constituents in Juglans regia L. peeled kernels and pellicles, using HPLC–MS/MS. Food Chem. 352, 129404. doi: 10.1016/j.foodchem.2021.129404 33676122

[B28] Medic A. Jakopič J. Solar A. Hudina M. Veberic R. (2021/a). Walnut (J. regia) agro-residues as a rich source of phenolic compounds. Biology 10 (6), 535. doi: 10.3390/biology10060535 34203814 PMC8232793

[B30] Mendelné Pászti E. Bujdosó G. Ercisli S. Hrotkó K. Mendel Á. (2023). Apricot rootstocks with potential in Hungary. Horticulturae 9, 720. doi: 10.3390/horticulturae9060720

[B31] Mystkowska I. Zarzecka K. Gugała M. Sikorska A. (2022). Profitability of using herbicide and herbicide with biostimulants in potato production. J. Ecol. Engineering. 23, 223–227. doi: 10.12911/22998993/146687

[B32] Nyitrainé Sárdy Á.D. Ladányi M. Varga Z. Szövényi Á.P. Matolcsi R. (2022). The effect of grapevine variety and wine region on the primer parameters of wine based on ^1^H NMR-spectroscopy and machine learning methods. Diversity 14, 74. doi: 10.3390/d14020074

[B33] Ombódi A. Toók B. (2022). Biostimulátor kezelés hatása szabadföldi paprikatermesztésben különböző indítótrágyák alkalmazása esetén [Effects of biostimulant treatment in field pepper cultivation using different starter fertilizers]. Kertgazdaság 54 (3), 51–62.

[B34] Özcan A. Sutyemez M. Bükücü Ş. (2022). Kurtulus 100, a new superior walnut cultivar in Turkey; field experimental comparative results with chandler. Erwerbs-Obstbau 65, 1–7. doi: 10.1007/s10341-022-00673-y

[B35] Paunović M. Rade C. (2023). Fruit characteristics of promising walnut genotypes from the region of eastern Serbia. Genetika-Belgrade 55, 193–202. doi: 10.2298/GENSR23010193P

[B36] Persic M. Mikulic-Petkovsek M. Halbwirth H. Solar A. Veberic R. Slatnar A. (2018/a). Red walnut: characterization of the phenolic profiles, activities and gene expression of selected enzymes related to the phenylpropanoid pathway in pellicle during walnut development. J. Agr. Food Chem. 66 (11), 2742–2748. doi: 10.1021/acs.jafc.7b05603 29494766

[B37] Peršić M. Mikulic-Petkovsek M. Slatnar A. Solar A. Veberic R. (2018/b). Changes in phenolic profiles of red-colored pellicle walnut and hazelnut kernel during ripening. Food Chem. 252, 349–355. doi: 10.1016/j.foodchem.2018.01.124 29478553

[B38] Petrova I. Ivanova S. Stoyanova S. Mincheva R. Pavlova M. (2023). Influence of biostimulants and humic extracts treatment on the fatty acid profile of the spring oilseed rape variety. Agric. Sci. Technology. 15, 52–59. doi: 10.15547/ast.2023.01.006

[B39] Popovic B. Engler M. Kovačić Đ. Herman G. Bukvić G. Ergović L. (2022). The influence of biostimulants on strawberries yield, nutritional and sensory fruit quality [Utjecaj biostimulanta na prinos, hranjivu i senzornu kvalitetu plodova jagoda.]. Glasnik zaštite bilja. 45, 84–89. doi: 10.31727/gzb.45.6.8

[B40] Rahmani F. Dehganiasl M. Heidari R. Rezaee R. Darvishzadeh R. (2018). Genotype impact on antioxidant potential of hull and kernel in Persian walnut (Juglans regia L.). Int. Food Res. J. 25, 35–42.

[B41] Rodriguez-Rajo F. J. Frenguelli G. Ato M. V. (2003). Effect of air temperature on forecasting the start of the Betulapollen season at two contrasting sites in the south of Europe(1995–2001). Int. J.Biometeorol. 47, 117–125. doi: 10.1007/s00484-002-0153-z 12748841

[B42] Shen D. Yuan X. Zhao Z. Wu S. Liao L. Tang F.. (2021). Determination of phenolic compounds in walnut kernel and its pellicle by ultra-high-performance liquid chromatography-tandem mass spectrometry. Food Anal. Method. 14. doi: 10.1007/s12161-021-02069-2

[B43] Sheng F. Hu B. Y. Jin Q. Wang J. B. Wu C. Luo Z. (2021). The analysis of Phenolic Compounds in walnut Huskand Pellicle by UPLC-Q-Orbitrap HRMS and HPLC. Molecules 26, 3013. doi: 10.3390/molecules26103013 34069333 PMC8158686

[B44] Slatnar A. Mikulic-Petkovsek M. Stampar F. Veberic R. Solar A. (2015). Identification and quantification of phenolic compounds in kernels, oil and bagasse pellets of common walnut (Juglans regia L.). Food Res. Int. 67, 255–263. doi: 10.1016/j.foodres.2014.11.016 30011716

[B45] Sokolova V. (2022). Selection of a breeding source material in the collection of the walnut (Juglans regia L.) of the Main Botanical Garden RAS. Pomiculture small fruits culture Russia. 70, 7–18. doi: 10.31676/2073-4948-2022-70-7-18

[B47] Sutyemez M. Özcan A. Yılmaz A. Yildirim E. Bükücü Ş. (2022). Determining phenological and genetic variation in genotypes obtained from open-pollinated seeds of ‘Maraş 12’ walnut (Juglans regia L.) cultivar. Genet. Resour. Crop E. 69, 823–838. doi: 10.1007/s10722-021-01267-5

[B50] Trandafir I. Cosmulescu S. (2020). Total phenolic content, antioxidant capacity and individual phenolic compounds of defatted kernel from different cultivars of walnut. Erwerbs-Obstbau 62, 1–6. doi: 10.1007/s10341-020-00501-1

[B49] Trandafir I. Cosmulescu S. Botu M. Nour V. (2016/b). Antioxidant activity, and phenolic and mineral contents of the walnut kernel ( Juglans regia L.) as a function of the pellicle color. Fruits 71, 177–184. doi: 10.1051/fruits/2016006

[B48] Trandafir I. Cosmulescu S. Nour V. (2016/a). Phenolic profile and antioxidant capacity of walnut extract as influenced by the extraction method and solvent. Int. J. Food Eng. 13. doi: 10.1515/ijfe-2015-0284

[B51] Vahdati K. Arab M. Sarikhani S. Sadat-Hosseini M. Leslie C. Brown P. (2019). ““Advances in Persian Walnut (Juglans regia L.) Breeding Strategies”,” in Advances in Plant Breeding Strategies: Nut and Beverage Crops. Eds. Al-Khayri J. Jain S. Johnson D. (Dordrecht, Germany: Springer), 401–472. doi: 10.1007/978-3-030-23112-5_11

[B52] Wang R. Tian X. Li Q. Liao L. Wu S. Tang F.. (2022). Walnut pellicle color affects its phenolic composition: Free, esterified and bound phenolic compounds in various colored-pellicle walnuts. J. Food Compos. Anal. 109, 104470. doi: 10.1016/j.jfca.2022.104470

[B58] Website 1: Website of Kwizda (2023). Available at: https://kwizda.hu/wuxal-ascofol~p13394 (Accessed 1st June 2023).

[B59] Website 2: Website of Kwizda Garden (2023). Available at: https://kwizdagarden.hu/professzionaliskerteszet/termekek (Accessed 1th June 2023).

[B60] Website 3:Website of Huminis Ltd. Available at: https://huminisz.hu/project/kondisol/ (Accessed 1th June 2023).

[B53] Wielgolaski F. E. (2001). Phenological modifications in plants byvarious edaphic factors. Int. J. Biometeorol. 45, 196–202. doi: 10.1007/s004840100100 11769320

[B54] Wielgolaski F. E. (2003). Climatic factors governing plant pheno-logical phases along a Norwegian fjord. Int. J. Biometeorol. 47, 213–220. doi: 10.1007/s00484-003-0178-y 12750970

[B56] Wu S. Mo R. Wang R. Li Q. Shen D. Liu Y. (2023). Identification of key antioxidants of free, esterified, and bound phenolics in walnut kernel and skin. Foods 12, 825. doi: 10.3390/foods12040825 36832900 PMC9956992

[B55] Wu S. Shen D. Wang R. Li Q. Mo R. Zheng Y.. (2021). Phenolic profiles and antioxidant activities of free, esterified and bound phenolic compounds in walnut kernel. Food Chem. 350, 129217. doi: 10.1016/j.foodchem.2021.129217 33607410

[B57] Zsigrai GY. Juhász CS. (2000). Különböző ökológiai K- és Mn-lombtrágya készítmények hatásának összehasonlító vizsgálata a Tokaji Borvidéken: - a fürttömeg, a mustminőség és a levélnyél kémiai összetételének változásai [Comparative study of different organic K and Mn fertilizers on the claster weight, quallity of juice, and leaf stalk in Tokaj wine region] in Hungarian. Szőlő-Levél 10 (5), 32–49.

